# Bedside echocardiography to predict mortality of COVID-19 patients beyond clinical data: Data from the PROVAR-COVID study

**DOI:** 10.1590/0037-8682-0382-2021

**Published:** 2021-09-06

**Authors:** Sander Luis Gomes Pimentel, Bruno Ramos Nascimento, Juliane Franco, Kaciane Krauss Bruno Oliveira, Clara Leal Fraga, Frederico Vargas Botinha de Macedo, Leonardo Arruda de Moraes Raso, Renata Eliane de Ávila, Luiza Pereira Afonso dos Santos, Rodrigo Tavares Lanna Rocha, Renan Mello Oliveira, Márcia de Melo Barbosa, Craig Sable, Antonio Luiz Pinho Ribeiro, Andrea Zawacki Beaton, Maria Carmo Pereira Nunes

**Affiliations:** 1 Universidade Federal de Minas Gerais, Centro de Telessaúde do Hospital das Clínicas, Serviço de Cardiologia e Cirurgia Cardiovascular, Belo Horizonte, MG, Brasil.; 2 Universidade Federal de Minas Gerais, Faculdade de Medicina, Departamento de Clínica Médica, Belo Horizonte, MG, Brasil.; 3 Hospital Eduardo de Menezes, Serviço de Infectologia, Belo Horizonte, MG, Brasil.; 4 Cardiology, Children’s National Health System, Washington, DC, United States of America,; 5 The Heart Institute, Cincinnati Children’s Hospital Medical Center, Cincinnati, OH, United States of America.

**Keywords:** COVID-19, Echocardiography, Prognosis, SARS-Cov-2, Mortality

## Abstract

**INTRODUCTION:**

Cardiac involvement seems to impact prognosis of COVID-19, being more frequent in critically ill patients. We aimed to assess the prognostic value of right ventricular (RV) and left ventricular (LV) dysfunction, evaluated by bedside echocardiography (echo), in patients hospitalized with COVID-19.

**METHODS:**

Patients admitted in 2 reference hospitals in Brazil from Jul to Sept/2020 with confirmed COVID-19 and moderate/severe presentations underwent clinical and laboratory evaluation, and focused bedside echo (GE Vivid-IQ), at the earliest convenience, with remote interpretation. The association between demographics, clinical comorbidities and echo variables with all-cause hospital mortality was assessed, and factors significant at p<0.10 were put into multivariable models.

**RESULTS:**

Total 163 patients were enrolled, 59% were men, mean age 64±16 years, and 107 (66%) were admitted to intensive care. Comorbidities were present in 144 (88%) patients: hypertension 115 (71%), diabetes 61 (37%) and heart failure 22 (14%). In-hospital mortality was 34% (N=56). In univariate analysis, echo variables significantly associated with death were: LV ejection fraction (LVEF, OR=0.94), RV fractional area change (OR=0.96), tricuspid annular plane systolic excursion (TAPSE, OR=0.83) and RV dysfunction (OR=5.3). In multivariate analysis, after adjustment for clinical and demographic variables, independent predictors of mortality were age≥63 years (OR=5.53, 95%CI 1.52-20.17), LVEF<64% (OR=7.37, 95%CI 2.10-25.94) and TAPSE<18.5 mm (OR=9.43, 95% CI 2.57-35.03), and the final model had good discrimination, with C-statistic=0.83 (95%CI 0.75-0.91).

**CONCLUSION:**

Markers of RV and LV dysfunction assessed by bedside echo are independent predictors of mortality in hospitalized COVID-19 patients, after adjustment for clinical variables.

## INTRODUCTION

The coronavirus disease 2019 (COVID-19) pandemic, caused by widespread infection with severe acute respiratory syndrome coronavirus 2 (SARS-CoV-2), has had a striking impact on Brazilian health, with almost 8 million cases and > 200,000 deaths occurring through January 2021^1^. Aside from the absolute number of cases and deaths directly attributable to the virus, the excess mortality—a metric to additionally evaluate the indirect impacts of the disease—was also remarkable in this period, including that associated with cardiovascular disease (CVD)^2^. 

There are ongoing investigations aimed at defining prognostic factors for patients with COVID-19, and risk factors (e.g., hypertension, diabetes, obesity), biomarkers (e.g., troponins, D-dimer, inflammatory markers), in addition to heart failure and disease-related cardiac disease, are associated with poor outcomes^3^ ^,^ ^4^. Echocardiography (echo) has emerged as a promising modality to improve outcome prediction in those with COVID-19, being simple, applicable at the bedside, and with a lower risk for contamination^5^. Echo-detected cardiac abnormalities are frequent in individuals with COVID-19. In a series involving > 1200 patients, 667 (55%) had abnormal echo results, with left (LV) and right ventricular (RV) abnormalities reported in 39% and 33%, respectively, and evidence of new myocardial infarction in 3%^6^. In addition, markers of RV dysfunction are associated with biomarkers of worse prognosis, such as troponin and D-dimer, and have been proposed as independent predictors of all-cause mortality^7^ ^,^ ^8^. Echo screening for acute heart failure has been proposed for critically ill COVID-19 patients in international position statements^9^. 

The mechanisms underlying these findings, however, are still under investigation. It is known that COVID-19 affects the cardiovascular system through different pathways, including direct myocardial injury due to viral invasion, systemic inflammatory response, and excessive cytokine release, resulting in multiple organ injury, plaque rupture, and thrombosis, and increased cardiometabolic demand^10^ ^-^ ^12^. Acute cardiac disease occurs in 8% to 20% of all COVID-19 patients^10^ ^-^ ^13^.

Thus, prospective systematic studies investigating the impact of routine echo assessments are of the utmost importance in the context of the COVID-19 pandemic. In this study, we aimed to assess the prognostic value of several echocardiographic variables, evaluated using bedside echo, in patients with COVID-19 in Brazil who fulfilled the criteria for hospitalization.

## METHODS

The PROVAR+ program has been conducted since 2017 in the state of Minas Gerais, southeastern Brazil, under the auspices of the *Universidade Federal de Minas Gerais* and the Telehealth Network of Minas Gerais^14^, in collaboration with the Children’s National Health System, Washington, DC, USA, and the Cincinnati Children’s Hospital Medical Center, Cincinnati, OH, USA. The program uses portable and ultra-portable echo devices for imaging acquisition and remote interpretation by telemedicine in different settings. The study protocol conformed to the ethics guidelines of the 1975 Declaration of Helsinki, and ethics approval was obtained from the Institutional Review Boards of *Universidade Federal de Minas Gerais* and *Hospital Eduardo de Menezes*. Data analytic methods and study materials will be made available to other researchers for purposes of reproducing the results or replicating the protocol from the corresponding author upon reasonable request. 

In this study, consecutive patients hospitalized in two reference hospitals (*Hospital Eduardo de Menezes, Fundação Hospitalar do Estado de Minas Gerais*, Belo Horizonte, MG, Brazil and *Hospital das Clínicas da Universidade Federal de Minas Gerais*, Belo Horizonte, MG, Brazil) with confirmed COVID-19 (positive real-time polymerase chain reaction [RT-PCR] for SARS-CoV-2) and a clinical spectrum of moderate and severe clinical presentations according to the Berlin definition^15^, as defined by the medical staff, underwent a standardized and detailed clinical questionnaire and laboratory evaluation, including complete blood count, serology for COVID-19 (immunoenzymatic assay, immunoglobulin [Ig]M and IgG), and biochemistry (including liver and renal function tests, and inflammatory markers), analysed in central laboratories. All patients underwent bedside echocardiography (Vivid IQ and Vivid Q, GE Healthcare, Milwaukee, WI, USA), with acquisition of standard parasternal and apical views by experienced cardiologists (SP, MCN, LMR) with local support from the study staff (nurses, physical therapists, medical students) for further remote consensus interpretation (MMB, MCN). Examinations were performed at the earliest convenience following admission, considering clinical and technical issues to acquire interpretable images and medical priorities defined by the intensive care staff. 

Echocardiographic images were uploaded to a dedicated reading system (EchoPAC®, GE Healthcare) and reviewed by two expert cardiologists (MN and MMB), and discrepancies were consensually solved. The comprehensive echocardiographic protocol focused on LV and RV morphology and function, mitral, aortic and tricuspid valves, pulmonary artery pressure and morphology, segmental wall motion abnormalities, and pericardial effusion^14^. Echocardiographic measurements were performed offline, according to the recommendations of the American Society of Echocardiography^16^ ^-^ ^18^. Global RV function was quantitatively assessed using fractional area change, peak systolic velocity at the tricuspid annulus using tissue Doppler imaging, and tricuspid annular plane systolic excursion (TAPSE) at the RV free wall, obtained using two-dimensional (2D) guided M-mode recordings. Systolic pulmonary artery pressure was measured using the tricuspid regurgitant velocity. Diastolic function was assessed using pulsed-wave Doppler examination of mitral inflow and tissue Doppler imaging. Left ventricular ejection fraction (LVEF) was calculated using the modified Simpson methodor, in cases with poor images, by linear dimensions with 2D-guided measurements^16^ ^-^ ^18^. Objective and subjective observations were also reported. Preliminary reports were promptly made available to the hospital for clinical care. The main COVID-19 unit (*Eduardo de Menezes*), a public hospital operated by the State Board of Health, has very limited access to imaging and relies largely on echo screening for medical decisions, while the *Hospital das Clínicas* is a quaternary university hospital with local computed tomography and magnetic resonance imaging availability.

All data were entered into a RedCap database^19^. Statistical analysis was performed using SPSS version 23.0 (IBM Corporation, Armonk, NY, USA) for Mac OSX (Apple Inc, Cupertino, CA, USA). Considering preliminary studies that suggested that approximately 20% of the population infected with SARS-CoV-2 present some degree of RV involvement, a 95% power and alpha error of 5%, a minimum sample of 143 patients was calculated. Considering losses and data completeness issues, consecutive eligible patients admitted over a two-month period (July and August 2020) were included during the first peak of the pandemic in Belo Horizonte, Brazil. The primary outcome was all-cause in-hospital mortality. Data are presented for all patients with RT-PCR-confirmed COVID-19. Categorical variables, expressed as number and percentage, were compared between groups (patients who recovered versus [vs.] those who died in the index hospitalization) using Fisher’s exact test, whereas continuous data, expressed as mean ± standard deviation (SD) or median (interquartile range [IQR] i.e., 25%-75%), were compared using the Student’s unpaired *t*-test or the Mann-Whitney U test, as appropriate. The association between demographic variables (age and sex), clinical comorbidities, and echocardiographic variables (Table 1) and the primary outcome was assessed using univariate logistic regression analysis. Factors significant at p < 0.10 were entered into the multivariable adjusted logistic models. Significant continuous variables in the final model were dichotomized, with optimal cut-offs defined from receiver operating characteristic (ROC) curves, and a new simplified model was adjusted. Differences with a two-tailed p ≤ 0.05 were considered to be statistically significant. 

## RESULTS

A total of 163 patients (96 [59%] male; mean age 64 ± 16 years) were consecutively enrolled, of whom 107 (66%) had severe COVID-19 with admission to intensive care during the index hospitalization. The median time from symptom onset to admission was 7 days (range 4.0-9.0) days. Demographic, clinical, and echocardiographic characteristics of the study sample are summarized in Table 1. During hospitalization, 56 patients died, corresponding to an overall mortality rate of 34%. 

Clinical comorbidities were present in 144 (88%) patients, with hypertension in 115 (71%), diabetes in 61 (37%) and heart failure in 22 (14%), and were similar among those who died and survived in the index hospitalization. However, patients who recovered were significantly younger (Table 1). The most frequently reported symptoms at admission were dyspnea (n = 132 [81%]), cough (n =122 [75%]), and fever (n = 99 [61%]). Dyspnea was more frequently reported by patients who died and, conversely, taste loss was more prevalent among those with favorable outcomes (Table 1).

**FIGURE 1:**
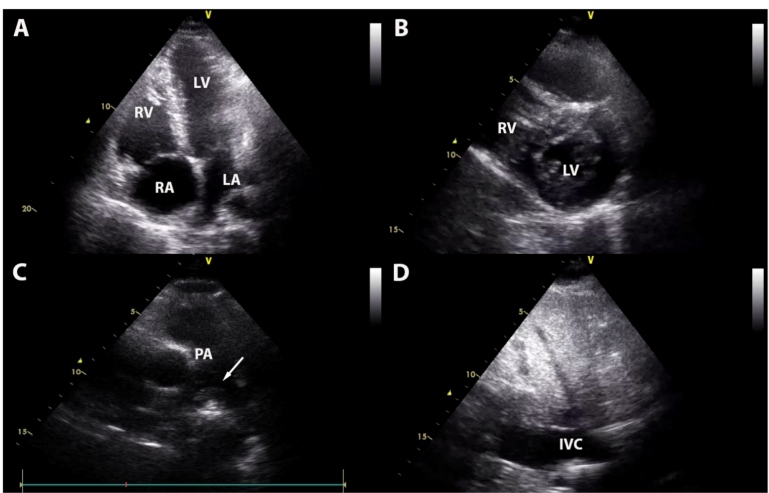
Echocardiographic images of a young (42 years-old) male patient who evolved with severe COVID-19, showing RV enlargement **(A)** with a D-shaped LV **(B)**, a proximal mobile thrombus in the main pulmonary artery **(C)** and a dilated IVC **(D)**. This patient died during the index hospitalization. **IVC:** inferior vena cava; **LA:** left atrium; **LV:** left ventricle; **PA:** pulmonary artery; **RA:** right atrium; **RV:** right ventricle.

The clinical course of patients who died was also less favorable, with a higher proportion of individuals requiring intubation and mechanical ventilation (94.6% versus [vs.] 26.2%, respectively; p < 0.001) and administration of vasopressors (91.1% vs. 19.6%; p < 0.001) (Table 1). In addition, this group had a higher burden of abnormalities in bedside echo, especially those associated with LV and RV function and dimensions. Moderate and severe mitral regurgitation, however, was more prevalent among survivors (Table 1). Pulmonary artery thrombosis was directly diagnosed in one case, with a mobile thrombus in the pulmonary artery (Figure 1).

**TABLE 1:** Demographic, clinical and echocardiographic characteristics of patients who survived and died during the index hospitalization.

Variable:	Survivors (N=107)	Hospital death (N=56)	P-value:
**Demographics and risk factors:**			
Age (median, (IQR))	62.0 (52.2 - 71.5)	68.2 (61.8 - 78.8)	0.010*
Sex (male, N (%)):	63 (58.9)	33 (58.9)	0.402
Any clinical comorbidities (N, %)	93 (86.9)	51 (91.1)	0.608
Hypertension (N, %):	75 (70.1)	40 (71.4)	1.000
Diabetes (N, %):	36 (33.6)	25 (44.6)	0.217
Heart failure (N, %):	15 (14.1)	7 (12.5)	0.812
Obesity (N, %):	13 (12.1)	5 (8.9)	0.786
Asthma (N, %):	5 (4.7)	4 (7.1)	0.721
Smoking (N, %):	26 (24.3)	13 (23.2)	1.000
**COVID-19 symptoms at admission:**			
Symptom onset to admission (days, median (IQR))	7.0 (4.0 - 9.0)	7.0 (5.0 - 11.0)	0.432
Fever at admission (>37.8 ^o^C) (N, %):	65 (60.7)	34 (60.7)	0.739
Cough (N, %):	80 (74.8)	42 (75.0)	1.000
Dyspnea (N, %):	80 (74.7)	52 (92.9)	0.006*
Diarrhea (N, %):	19 (17.8)	4 (7.1)	0.096
Headache (N, %)	14 (13.1)	3 (5.4)	0.269
Abdominal pain (N, %):	5 (4.7)	3 (5.4)	1.000
Anosmia (N, %):	17 (15.9)	3 (5.4)	0.121
Taste loss (N, %):	18 (16.8)	2 (3.6)	0.036*
**Respiratory support and vasopressors:**			
Noninvasive ventilation (N, %)	14 (13.1)	10 (17.9)	0.486
Mechanical ventilation (N, %)	28 (26.2)	53 (94.6)	<0.001*
Days in mechanical ventilation (median, (IQR))	13.0 (6.0 - 23.0)	10.0 (7.0 - 16.0)	0.525
Tracheostomy (N, %)	10 (9.3)	15 (26.8)	0.618
Use of vasopressors (N, %)	21 (19.6)	51 (91.1)	<0.001*
**Echo variables:**			
Admission to echo (days, median (IQR))	6.0 (3.0 - 9.0)	7.0 (3 - 12)	0.200
LVEF (%)	65.0 (61.0 - 70.0)	61.0 (46.0 - 66.0)	<0.001*
LV diastolic diameter (mm, median (IQR))	45.0 (43.0 - 49.0)	45.0 (40.0 - 49.0)	0.212
LV systolic diameter (mm, median (IQR))	29.0 (26.0 - 32.0)	32 (28.0 - 36.0)	0.008*
RV dysfunction (any) (N, %)	11 (10.3)	21 (37.5)	<0.001*
LA diameter (mm) (mean±SD)	37.9±4.7	36.3±6.1	0.084
RV fractional area change (%)(mean±SD)	39.8±10.0	35.6±11.4	0.046*
TAPSE (mm, median (IQR))	20.0 (18.0 - 23.0)	16.0 (14.0 - 19.0)	<0.001*
RV basal dimension (mm, median (IQR))	34.0 ( 31.0 - 38.0)	38.0 (36.0 - 43.0)	<0.001*
Septal E/E’ (median (IQR))	9.3 (8.0 - 11.8)	10.3 (9.2 - 13.0)	0.079
Tricuspid velocity (m/s) (mean±SD)	2.8±0.4	3.0±0.4	0.188
Mitral regurgitation (moderate/severe) (N, %):	12 (11.2)	1 (1.8)	0.007*
LV wall-motion abnormalities (N, %):	7 (6.5)	6 (10.7)	0.369
Pericardial effusion (N, %):	15 (14.0)	10 (17.8)	0.493

**Abbreviations: LA:** left atrium; **LV:** left ventricle; **LVEF:** left ventricular ejection fraction; **OR:** odds-ratio; **RV:** right ventricle; **TAPSE:** tricuspid annular plane systolic excursion. *p<0,05.

**FIGURE 2:**
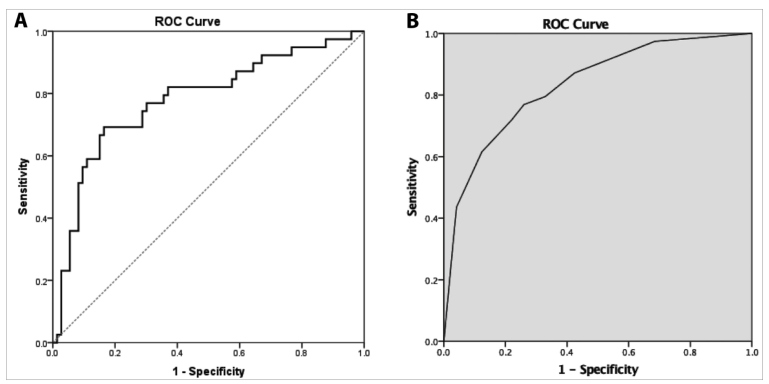
**A:** Receiver Operating Characteristic (ROC) curve of the model including the significant variables in the multivariate logistic model to predict in-hospital mortality for patients with COVID-19; C-statistic = 0.78 (95% CI 0.68 - 0.88). **B:** ROC curve of the simplified model with dichotomized predictors of in-hospital mortality (age ≥63 years, LVEF <64% and TAPSE <18.5 mm); C-statistic = 0.83 (95% CI 0.75 - 0.91).

On univariate analysis, echocardiographic RV and LV function variables were significantly associated with death: LVEF (odds ratio [OR] 0.94 [95% confidence interval [CI] 0.92-0.98); RV fractional area change (OR 0.96 [95% CI 0.93-0.99]); TAPSE (OR 0.83 [95% CI 0.74-0.93]) and any degree of RV dysfunction (OR 5.33 [95% CI 2.33-12.21)’; as well as age (hazard ratio [HR] 1.03 [95% CI 1.01-1.05), dyspnea at presentation (OR 4.39 [95% CI 1.45-13.29), and taste loss (OR 0.21 [95% CI 0.05-0.97]) (Table 2). On multivariate analysis, the final model demonstrated good overall discrimination, with a C-statistic of 0.78 (95% CI 0.68-0.88) (Figure 2A). After adjustment for clinical and demographic variables, independent predictors of mortality included age (OR 1.05 [95% CI 1.01-1.10; p = 0.023), LVEF (OR 0.95 [95% CI 0.91-0.99]; p = 0.048) and TAPSE (OR 0.76 [95% CI 0.63-0.91]; p = 0.005) (Table 2). From the ROC curves, the optimal cut-offs and adjusted ORs for the significant variables included: age ≥ 63 years (OR 5.53 [95% CI 1.52-20.17]), LVEF < 64% (OR 7.37 [95% CI 2.10-25.94) and TAPSE < 18.5 mm (OR 9.43 [95% CI 2.57-35.03]); the final simplified prediction model yielded a C-statistic of 0.83 (95% CI 0.75-0.91) (Figure 2B). 

**TABLE 2:** Univariable and multivariable analysis of demographic, clinical and echocardiographic variables associated with in-hospital mortality.

Univariable analysis:			
Variable	OR:	95% CI:	P-value:
**Demographics and risk factors:**			
Age (each 1 year):	1.03	1.01 - 1.05	0.013*
Sex (male):	1.41	0.72 - 2.75	0.312
Hypertension:	0.98	0.41 - 2.28	0.926
Diabetes:	1.58	0.79 - 3.17	0.194
Heart failure:	0.85	0.32 - 2.24	0.737
Obesity:	0.73	0.24 - 2.24	0.587
Asthma:	1.50	0.38 - 5.85	0.561
Smoking:	0.94	0.44 - 2.02	0.877
**COVID-19 symptoms at admission:**			
Fever:	0.89	0.46 - 1.72	0.732
Cough:	1.09	0.51 - 2.33	0.823
Dyspnea:	4.39	1.45 - 13.29	0.009*
Diarrhea:	0.80	0.11 - 1.13	0.080
Abdominal pain:	1.16	0.27 - 5.07	0.840
Anosmia:	0.35	0.10 - 1.28	0.112
Taste loss:	0.21	0.05 - 0.97	0.045*
**Echo variables:**			
LVEF (%)	0.94	0.92 - 0.98	<0.001*
RV dysfunction (any)	5.33	2.33 - 12.21	<0.001*
LA diameter (mm)	0.94	0.88 - 1.00	0.062
RV fractional area change (%)	0.96	0.93 - 0.99	0.049*
TAPSE (mm)	0.83	0.74 - 0.93	0.001*
RV basal dimension (mm)	1.10	1.03 - 1.18	0.004*
Septal E/E’	1.07	0.97 - 1.17	0.202
Tricuspid velocity (m/s)	2.60	0.68 - 9.92	0.161
Multivariable analysis†:			
Age (each 1 year):	1.05	1.01 - 1.10	0.023*
LVEF (%):	0.96	0.91 - 0.99	0.048*
TAPSE (mm)	0.76	0.63 - 0.91	0.005*

**Abbreviations: LA:** left atrium; **LVEF:** left ventricular ejection fraction; **OR:** odds-ratio; **RV:** right ventricle; **TAPSE:** tricuspid annular plane systolic excursion. *p<0.05. † Multivariable analysis adjusted for sex, hypertension, diabetes, heart failure, asthma, obesity, fever, cough.

## DISCUSSION

Our data, obtained from a sample of inpatients with moderate and severe COVID-19 in Brazil, suggest that echocardiographic assessment performed at the bedside may be useful as a prognostic tool to improve early outcome prediction during the pandemic. Markers of RV dysfunction, combined with LVEF, may be used to predict mortality in addition to known clinical predictors. Being practical and easily applicable, comprehensive bedside echo may improve the access to early cardiac imaging, especially in resource-limited institutions.

Since the onset of the COVID-19 pandemic, and the high mortality observed in the elderly and in patients with significant clinical comorbidities^20^ ^,^ ^21^, efforts have been made to define independent predictors of adverse and fatal outcomes, as well as to refine prediction models. Published data indicate that patients with preexisting CVD are at highest risk for complications^20^, and resemble findings from previous epidemics, such as the SARS‐CoV and Middle Eastern Respiratory Syndrome coronavirus outbreaks, in which the association between pre-existing CVD and disease-induced myocardial injury with worse outcomes was observed^22^. Thus, there appears to be a superposition of mechanisms directly linked to viral infection (e.g., cardiomyopathy induced by coronaviruses in animal models^23^), the stress of infection, and a prothrombotic state, potentially inducing rapid decompensation in patients with heart failure or coronary artery disease^24^. In this sense, our data reinforce these assumptions, highlighting the impact of ventricular impairment, especially the RV.

Our sample consisted of moderate and severe cases requiring hospital admission and medical care (66% intensive care); as such, these patients are a platform to look deeper into different aspects of the disease. Regardless of whether COVID-19 exacerbates preexisting CVD or causes new structural/functional abnormalities^24^, early detection of such conditions may be valuable for patient management. Several symptoms of COVID-19, such as chest pain and marked dyspnea, overlap with those of acute CVD^25^ ^,^ ^26^, and observational data suggest that electrocardiographic abnormalities and elevated biomarkers of cardiac injury are also prevalent, especially in severely ill individuals^20^ ^,^ ^24^ ^,^ ^27^. This, along with the high cardiovascular risk profile of hospitalized individuals, as evidenced in our sample, may trigger requests for echo in a large majority of patients. In institutions with limited imaging capabilities, such as one of our COVID-19 centers, this indication is even more frequent in the absence of a more advanced propaedeutic. Considering this, incorporation of echo in the management of COVID-19 patients who require hospital admission—regardless of underlying cardiac disease—into prognostic models is crucial.

On the other hand, despite the growing body of evidence supporting the utility of echo in COVID-19, ideal imaging protocols need to be evaluated considering the shortage of personal protective equipment and the exposure of personnel. Considering the high-risk setting, especially in the intensive care environment, we opted to apply a fast-track comprehensive protocol. Although simplified to optimize screening time, it enabled a more accurate cardiac assessment compared with those proposed in recently published studies^8^ ^,^ ^28^. Thus, functional portable machines—and selected handheld devices—can be used^29^ to improve availability and reduce costs. Beyond limiting the exposure time of screeners, non-experts (i.e., clinical personnel already in contact with COVID-19 patients) may be trained to perform imaging acquisition, and telemedicine may enable remote interpretation. Similar equipment and strategies may be applied to point-of-care lung ultrasound (POCUS)^30^. Task shifting, however, requires a learning curve, and the echo variables of interest must be carefully evaluated in this case.

Our key findings, suggesting RV dysfunction as a predictor of death, align with preliminary data from China and the United Kingdom (UK)^8^ ^,^ ^31^; however, those studies reported relative LV sparing, in contrast to our observations. Regarding the choice of parameters for RV assessment, Li and Cols successfully demonstrated RV free-wall strain to be a more accurate predictor of unfavorable outcomes compared with other traditional variables^31^. This choice, however, conflicts with recommendations for simple and easily applicable level-1 protocols during the pandemic^32^ ^,^ ^33^. For this reason, we opted to systematically evaluate more practical and reproducible measures, as proposed by Moody and Argulian in the UK and the United States and supported by recent position statements^7^ ^,^ ^8^. As such, the RV variable independently associated with unfavorable outcomes in the present study was TAPSE, which has been previously correlated with acute-phase biomarkers such as troponins and D-dimer^8^. Moreover, RV dysfunction appears to independently predict death in COVID-19 patients, even after adjustment for D-dimer values^34^, with more recent data reinforcing its association with high-sensitivity troponin, one of the strongest predictors of in-hospital mortality^35^. The mechanisms behind this finding, however, remain controversial because RV dysfunction/enlargement may result from pulmonary vascular involvement and respiratory support. 

In a European study performed in hospital settings, pulmonary hypertension, but not RV dysfunction, was associated with the composite outcome of death and/or indication for intensive care^36^. The study population, however, exhibited much less severe presentations, with 4% requiring intubation and < 10% mortality, compared to > 66% intensive care admissions and 34% mortality in our sample^36^. Risk factors were also less prevalent. Thus, we hypothesize that infectious and inflammatory mechanisms presumably triggering RV impairment^8^ are more intensive, and baseline cardiac disease is more prevalent. In our study, the absence of elevated tricuspid velocity suggests mechanisms other than pulmonary hypertension to explain RV dysfunction. In fact, RV function is determined by intrinsic RV contractility and ventricular preload/afterload. Therefore, its dysfunction in the setting of COVID-19 is likely related to several mechanisms aside from elevated pulmonary artery pressure.

In addition, pulmonary pressure is multifactorial, with marked variations in response to hypoxemia-related vasoconstriction, increased positive end-expiratory pressure (PEEP), and other ventilator parameters^37^ being more prone to bias in prediction models. Although pulmonary thrombosis resulting from hypercoagulation has been described in COVID-19^36^, chest computed tomography and lung scintigraphy were not routinely performed in our patients, and proximal thrombus was observed in only one case (Figure 1). 

Regarding LV involvement observed in our study, it has been previously reported in other viral infections with intense systemic manifestations, such as Dengue^38^, Zika^39^, Chikugunya^40^, and yellow fever^41^, which are often associated with impaired systolic function. As with RV involvement, it remains unclear whether this results from direct viral infection or replication leading to cytokine dysregulation^42^—mechanisms involved in severe COVID-19^4^ ^,^ ^21^—or is derived from hemodynamic instability and shock decompensating underlying disease. Pathological evidence of myocarditis has also been reported^43^. As expected, a much higher proportion of patients with unfavorable outcomes in our study (the subset with lower LVEF) required hemodynamic support with vasopressors (Table 1). For both LVEF and TAPSE, the conservative cut-offs in our final dichotomous prediction model were presumably associated with the hyperdynamic state of patients with moderate and severe COVID-19, resulting in tachycardia and high output. 

In our sample, traditional cardiovascular risk factors for poor outcomes in epidemiological studies^4^ ^,^ ^21^ were not associated with mortality and were evenly distributed between survivors and those who died (Table 1). This may be due to our patient selection, consisting only of individuals requiring hospitalization with at least moderate symptoms, which may hinder the risk profile gradient between groups. For this reason, our model may not be generalizable to populations of mildly symptomatic patients. However, it is noteworthy that echo parameters remained as predictors even after adjusting for clinical variables. Finally, in addition to investigating predictive parameters, our program, with a fast-track imaging routine, improved access to healthcare because we prioritized screening for those who required immediate echo information, as indicated by the medical staff, which potentially facilitated lifesaving interventions in the short term. However, further studies examining the outcomes are warranted. With refinement of the model, this experience from a Latin American country can be replicated in other under-sourced regions.

## LIMITATIONS

Our study had several limitations. First, due to logistics at each hospital and the severity of cases, echos were performed at the earliest convenience following admission, and not at a standardized time point after severe symptoms presented. For this reason, the results may reflect different stages of systemic inflammation and cardiac involvement. Second, no serial examinations were performed, precluding definitive conclusions regarding the longitudinal progression/regression of cardiac abnormalities in the course of COVID-19, and the study time was fixed (period of hospitalization). Thus, the cross-sectional nature of the data limits more definitive conclusions regarding prognosis. Third, we opted to include patients with different degrees of oxygen supply and hemodynamic support, including those undergoing mechanical ventilation and/or vasopressor therapy at enrolment. Thus, hemodynamic responses to variable pulmonary pressures, including high PEEP and different patterns of systemic and pulmonary vascular resistance, may impact echo variables. Finally, because severely ill patients - who were sometimes transferred from pre-hospital emergency units - were included, clinical data were prone to imprecision, being frequently informed by relatives or companions. Data collection was pragmatic and, as a result, variables, such as Chagas disease, an endemic infectious condition in Brazil that may affect right heart chambers, were not systematically collected. However, our data, from a pioneer analysis involving a Latin American sample, reflects the real-life approach to patients with moderate to severe COVID-19, and the clinical variability may reflect the heterogeneity observed in the management of such cases. Our model suggests that, even with the aforementioned heterogeneity, bedside focused echo was an useful tool for risk stratification, with predictive value in addition to clinical presentation. 

## CONCLUSION

Markers of RV and LV dysfunction evaluated using focused bedside echo were independently associated with all-cause mortality in hospitalized COVID-19 patients after adjustment for clinical variables. Further studies using longitudinal data to confirm these findings are warranted. Echocardiographic assessment of ventricular function can be potentially helpful for clinical risk stratification early following admission of patients with COVID-19, being a simple and widely available tool during the pandemic, especially in resource-limited settings.

### Availability of data and material

Data analytic methods and study materials will be made available to other researchers for purposes of reproducing the results or replicating the protocol from the corresponding author upon reasonable request. 

### Ethics approval

The study protocol conformed to the ethical guidelines of the 1975 Declaration of Helsinki, and ethics approval was obtained from the Institutional Review Boards of *Universidade Federal de Minas Gerais and Hospital Eduardo de Menezes*.

### Consent to participate

All included patients provided informed consent before enrollment.

### Consent to publish

The corresponding author assures readers that all authors actively participated in this study. All of them have read and approved the manuscript submitted for publication, which reports unpublished work that is not under consideration elsewhere.
